# Molecular Mechanisms of Action of Emodin: As an Anti-Cardiovascular Disease Drug

**DOI:** 10.3389/fphar.2020.559607

**Published:** 2020-08-27

**Authors:** Qianqian Li, Jian Gao, Xiaohan Pang, Aiping Chen, Yi Wang

**Affiliations:** ^1^ School of Traditional Chinese Medicine, Beijing University of Chinese Medicine, Beijing, China; ^2^ College of Pharmaceutical Sciences, Pharmaceutical Informatics Institute, Zhejiang University, Hangzhou, China

**Keywords:** emodin, polyvalent molecule, mechanisms of action, targets identification, anti-cardiovascular disease

## Abstract

Emodin is a natural occurring anthraquinone derivative isolated from roots and barks of numerous plants, molds, and lichens. It is found to be an active ingredient in different Chinese herbs including *Rheum palmatum* and *Polygonam multiflorum*, and it is a pleiotropic molecule with diuretic, vasorelaxant, anti-bacterial, anti-viral, anti-ulcerogenic, anti-inflammatory, and anti-cancer effects. Moreover, emodin has also been shown to have a wide activity of anti-cardiovascular diseases. It is mainly involved in multiple molecular targets such as inflammatory, anti-apoptosis, anti-hypertrophy, anti-fibrosis, anti-oxidative damage, abnormal, and excessive proliferation of smooth muscle cells in cardiovascular diseases. As a new type of cardiovascular disease treatment drug, emodin has broad application prospects. However, a large amount of evidences detailing the effect of emodin on many signaling pathways and cellular functions in cardiovascular disease, the overall understanding of its mechanisms of action remains elusive. In addition, by describing the evidence of the effects of emodin in detail, the toxicity and poor oral bioavailability of mice have been continuously discovered. This review aims to describe a timely overview of emodin related to the treatment of cardiovascular disease. The emphasis is to summarize the pharmacological effects of emodin as an anti-cardiovascular drug, as well as the targets and its potential mechanisms. Furthermore, the treatment of emodin compared with conventional cardiovascular drugs or target inhibitors, the toxicity, pharmacokinetics and derivatives of emodin were discussed.

**Graphical Abstract:**
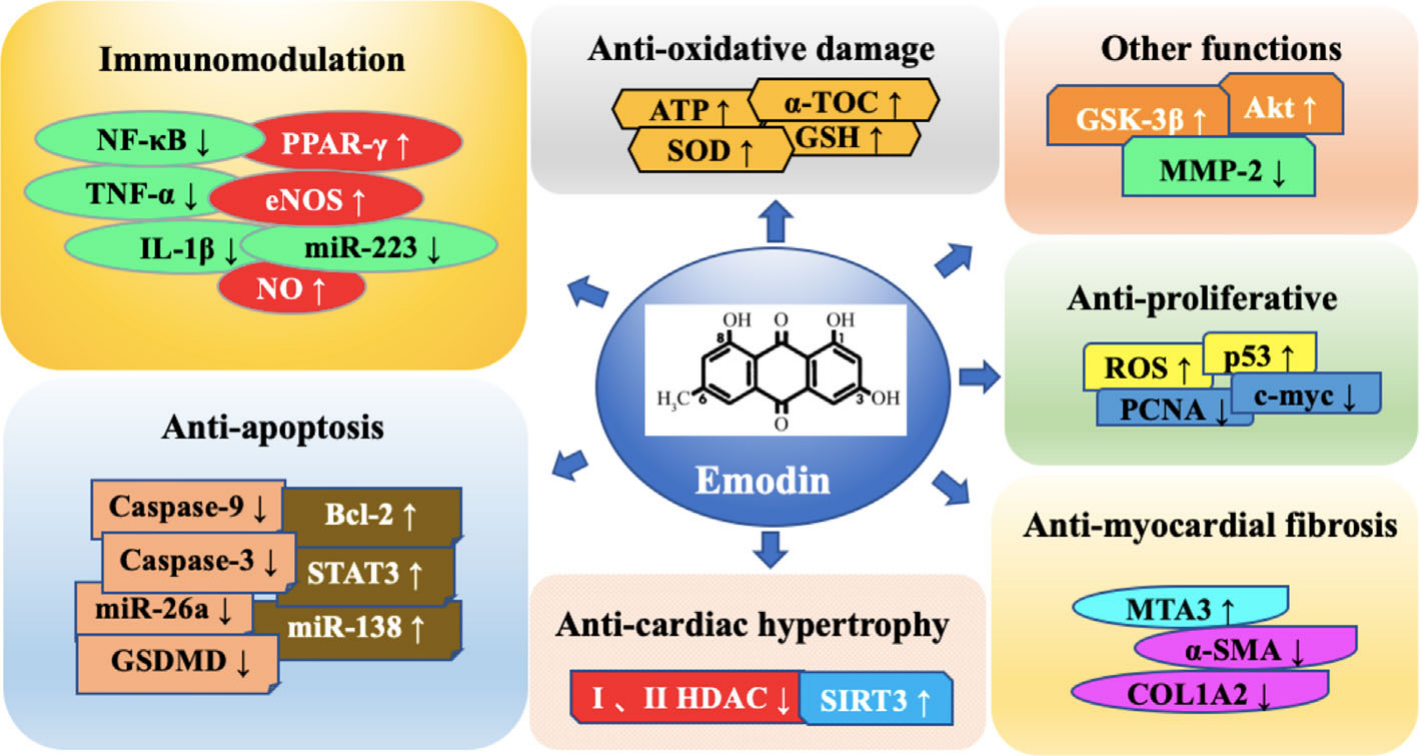


## Introduction

Emodin (1, 3, 8-trihydroxy-6-methylanthraquinone) is a natural anthraquinone derivative, and is the main ingredient of many herbs including *rhubarb, polygonum multiflorum*, and *polygonum cuspidatum* (Dong et al., 2016; Monisha et al., 2016). Pharmacological studies have shown that emodin, as a polyvalent molecule, has a variety of biological functions, such as anti-inflammatory (Li X. et al., 2020; Xu et al., 2020), anti-bacterial (Ji et al., 2020), anti-cancer (Wu C. et al., 2019; Ma et al., 2020; Rossi et al., 2020) and inhibition of oxidative stress(Lee et al., 2020). Emodin has also been shown to have potent anti-cardiovascular activities *in vitro* and *in vivo*, including anti-inflammatory (Wu et al., 2007), antioxidant damage (Du and Ko, 2005), inhibition of myocardial cell fibrosis (Evans et al., 2020) and other pharmacological effects. Based on these pharmacological effects, emodin has been shown to be functional in myocarditis, myocardial hypertrophy, ischemia-reperfusion injury, and myocardial infarction. This indicates that emodin plays an important role in the treatment of cardiovascular diseases (Shrimali et al., 2013). Numerous studies have also made huge strides in revealing the mechanisms behind these effects. In addition, comparable therapeutic effect and better assisting effect were also shown in the comparative study between emodin and conventional therapeutic drugs or target inhibitors (Du and Ko, 2005; Du and Ko, 2006; Liu et al., 2013; Evans et al., 2020). However, whether emodin has a specific therapeutic effect in cardiovascular diseases is still unclear, which limits its further research and application. In addition, although there is abundant evidence detailing the effects of emodin on various signaling pathways and cellular functions, the overall understanding of its mechanisms of action remains elusive. Therefore, the purpose of this paper is to provide an overview of emodin’s role in the fight against cardiovascular disease timely. The emphasis is to summarize the pharmacological effects of emodin as a possible anti-cardiovascular drug, as well as its targets and mechanisms of action.

## Targets and Studies of Emodin Against Anti-Cardiovascular Disease

Emodin has been shown to be active in a variety of cardiovascular diseases *in vivo* and *in vitro*. And numerous studies have explored the underlying mechanisms of these effects (**Table 1**).

**Table 1:** Pharmacological effects of emodin in anti-cardiovascular diseases.

References	Finding	Methodology
Immunomodulation		
(Wu et al., 2007)	TNF-α↓, NF-κB inhibited, caspase-3 inhibited	Emodin treatment in BALB/c mice with AMI
(Song et al., 2012)	TNF-α and IL-1β↓, NF-κBp65 inhibited	Emodin treatment in EAM in male Lewis rats
(Jiang et al., 2014)	IL-1β, IL-6, TNF-α, IL-23, and IL-17↓, NF-κBp65 inhibited	Emodin treatment in BALB/c mice with viral myocarditis
(Shou et al., 2018)	PPAR-γ and eNOS phosphorylation↑, NO↑	Emodin treatment in HAECs with ischemia-mimetic
(Xu et al., 2018b)	BMP2, TRAF1, and RELA↓, calcification, and phenotypical transformation of hVICs *via* the NF-κB signaling pathway	Emodin treatment in calcification of human aortic valve interstitial cells
(Yang et al., 2019)	IL-1β, IL-6, TNF-α, and↓, miR-223↓, cell viability, and cyclinD1↑, apoptosis was suppressed, Jnk signaling pathway inhibited by miR-223	Emodin treatment in H9c2 cells with myocarditis
Anti-apoptosis		
(Liu et al., 2013)	caspase-3↓, Bcl-2↑	Emodin treatment in BALB/c mice and HEp-2 cells with viral myocarditis
(Zhang et al., 2019)	miR-138↑, MLK3↓, p53 and p21↓, cyclin D1↑, caspase-3 and caspase-9↓Sirt1/AKT, and Wnt/β-catenin pathways activated,	Emodin treatment in H9c2 cells with myocardial ischemia
(Huang et al., 2019)	miR‐26a↓, survivin↑, caspase-3, and caspase-9↓, JAK1/STAT3 signal activated	Emodin treatment in H9c2 cells with myocardial ischemia
(Ye et al., 2019)	GSDMD-N↓, IL-1β↓, TLR4/MyD88/NF-κB/NLRP3 inhibited	Emodin treatment in Sprague-Dawley rats cardiomyocytes with ischemia/reperfusion injury
Anti-myocardial fibrosis		
(Xiao et al., 2019)	MTA3↑, COL1A2, and *α-*SMA↓	Emodin treatment in mouse model of pathological cardiac hypertrophy with excess fibrosis
Anti-cardiac hypertrophy		
(Evans et al., 2020)	I HDAC and II HDAC activity inhibited, histone acetylation in cardiomyocytes↑, ERK phosphorylation inhibited	Emodin treatment in C57BL/6 mice with cardiac hypertrophy (transverse aortic constriction-induced) and fibrosis (AngII-induced)
(Gao et al., 2020)	SIRT3↑, modulation of mitochondrial SIRT3 and its downstream signaling pathway	Emodin treatment or prevent in H9c2 cells with hypertrophy, primary cultured cardiomyocytes, and WT C57BL/6 mice and SIRT3-KO mice with cardiac hypertrophy
Anti-oxidative damage		
(Du and Ko, 2005)	ATP↑, myocardial mitochondrial SOD activity in female↑	Emodin pretreatment Sprague-Dawley isolated rat hearts (male and female) with I-R injury
(Du and Ko, 2006)	LDH leakage↓, mitochondrial antioxidant components↑, contractile force recovery↑	Emodin pretreatment Sprague-Dawley isolated rat hearts with I-R injury
Anti-proliferative		
(Wang X. et al., 2007)	damages DNA, production of ROS and expression of p53↑, VSMCs proliferation inhibited	Emodin treatment in abnormal proliferation and migration of VSMC
(Wang S. J. et al., 2008)	VSMCs proliferation inhibited	Emodin on VSMCs proliferation induced by Ang II
Other pathways and functions		
(Zhou et al., 2014)	ANP↑, L-type Ca (2+) channels inhibited, K (+) ATP channel activated	Emodin treatment in isolated perfused beating rabbit atria
(Chen et al., 2014)	MMP-2 and TIMP-2 expression↓	Emodin treatment in hypertensive rats with LV fibrosis in Goldblatt (2K1C)
(Wu et al., 2014)	Improve diabetes-induced systolic dysfunction. Akt and GSK-3β phosphorylation↑, HR↑, LVESD, LWPWT, and IVSD ↓.	Emodin treatment in Wistar rats with diabetic cardiomyopathy
(Nemmar et al., 2015)	Leukocytes, erythrocytes, hematocrit, and hemoglobin concentrations↓, IL-1β and TNF-α↓, antioxidant activities SOD and GR↑, shortening of the PT and aPTT reversed	Emodin treatment in Tuck-Ordinary mice with cardiac inflammation and oxidative stress

The results and potential mechanisms of cardiovascular protection are shown below.

### Immunomodulation

#### NF-κB

Inflammation is closely related to the occurrence of cardiovascular events (Della Corte et al., 2016; Del Pinto and Ferri, 2018). Abnormal secretion and expression of inflammatory factors are essential for the injury and necrosis of cardiovascular cells (Ait-Oufella et al., 2019). Emodin has been found to have effective anti-inflammatory effects (Shrimali et al., 2013; Ding et al., 2020), and it also has the activity in cardiovascular diseases. It is reported that emodin can control the expression and secretion of inflammatory factors by inhibiting the transcription factor nuclear factor kappa-B (NF‐κB) in a variety of inflammatory diseases. For example, emodin (30 mg/kg) significantly inhibited the expression of tumor necrosis factor-α (TNF-α) and the activation of NF-κB in the local myocardial infarction area of acute myocardial infarction (AMI) model mice, protected myocardial cell injury by inhibiting local inflammation, reduced the range of myocardial infarction induced by occluding the left anterior descending coronary artery in BALB/c mice in a dose-dependent manner (Wu et al., 2007). Similarly, emodin can regulate the inflammatory process in experimental autoimmune myocarditis (EAM) (50 mg/kg) and viral myocarditis (VMC) (0.3 mg) model mice by inhibiting the transcription factor NF‐κB, reducing the production of pro-inflammatory cytokines, such as interleukin-1β (IL-1β), interleukin-6 (IL-6), TNF-α, and decreasing the severity of myocarditis (Song et al., 2012; Jiang et al., 2014). Moreover, in lipopolysaccharide (LPS)-induced H9c2 cells (5, 10, and 15 μM of emodin), it has a similar effect to myocarditis (Yang et al., 2019). Emodin (10 μM) can also inhibit TNF-α-induced calcification and phenotypical transformation of human aortic valve interstitial cells (hVICs) *via* the NF-κB signaling pathway, thereby preventing calcification events stimulated in acute inflammatory responses (Xu et al., 2018b).

### PPAR-γ and eNOS

Abnormal expression of endothelial inflammatory cytokines and mediators is a critical factor leading to myocardial ischemia-reperfusion (I-R) injury (Sager and Nahrendorf, 2016). Peroxisome proliferator-activated receptor γ (PPAR-γ) is identified as a cardio-protective factor (Agrawal et al., 2014). I-R can inhibit the activation of PPAR-γ signaling pathway by reducing the phosphorylation of PPAR-protein (Xiong D. et al., 2016). PPAR-γ agonists can reduce I-R cell damage in multiple organs, including brain, liver, heart, and lung, and further activate endothelial nitric oxide synthase (eNOS), which plays a key role in maintaining and improving endothelial function (Kobayashi et al., 2008). In addition, eNOS inhibited the production of inflammatory cytokines by NO dependent mechanisms (Wang et al., 2017). Shou et al. (2018) established an *in vitro* hypoxia-reoxygenation model of human aortic endothelial cells (HAECs). The inactivation of PPAR-γ and eNOS signaling pathways is essential for the expression of inflammatory cytokines in endothelial cells induced by hypoxia-reoxygenation. The intervention of emodin reversed the levels of endothelial inflammatory cytokines and the expression of mediators in a concentration-dependent manner. Emodin recovered the PPAR-γ activity of hypoxia-reoxygenated endothelial cells by the molecular interaction between Heat Shock Proteins90 (HSP90) and eNOS. Therefore, it advances the production of NO, inhibits the inflammatory response, and has a protective effect on hypoxia-reoxygenation injury.

#### MiR-223

Studies have reported that MicroRNA (miR)-223 can be invoked as a new potential target for the diagnosis and treatment of inflammatory diseases, such as thrombophlebitis (Li H. et al., 2020), acute peritonitis (Brook et al., 2019), viral myocarditis (Gou et al., 2018), and acute lung injury (Feng et al., 2017). It can inhibit the inflammatory response and prevent the indirect damage in the process of infection (Yuan et al., 2018; Cavallari et al., 2019; Kim et al., 2019). Jun kinase enzyme (JNK) signaling regulates a wide range of cellular processes, including cell proliferation, differentiation, survival, apoptosis, and inflammation (Hammouda et al., 2020). (Yang et al. (2019) explored the effects of miR-223 and JNK signaling pathways on LPS-induced H9c2 cells, and found that LPS exposure significantly up-regulated the expression levels of miR-223 and JNK-related proteins (p-JNK and p-c-Jun), promoted apoptosis and the release of inflammatory factors IL-1β, IL-6, TNF-α. Furthermore, 20 μM of emodin intervention eliminated the upregulation. Emodin alleviated the LPS-induced myocarditis damage by inhibiting the over expression of miR-223. And by down-regulating miR-223 to inactivate the JNK signaling pathway. Therefore, miR-223 is considered as a possible target of emodin.

### Anti-Apoptosis

#### Caspase-3 and Caspase-9

Effector caspase-3 and the initiator caspase-9 have been recorded as important agents of apoptosis (Cao et al., 2002; Yao et al., 2007). Emodin has been repeatedly reported to down-regulate caspase-3 and caspase-9 due to myocardial cell injury *in vivo* and *in vitro* (Wu et al., 2007; Yang et al., 2019). Liu et al. (2013) first reported that the apoptosis rate of hep-2 cells treated with emodin was significantly lower than that of the control group infected with the virus. The 50% effective concentration (EC50) of the pro-apoptotic gene caspase-3 in the heart tissue of mice with coxsackievirus B4 (CVB4)-induced viral myocarditis down-regulated by emodin was 12.06 μM, and the selectivity index (SI) was 5.08. At the same time, the apoptosis inhibitor gene bcl-2 was up-regulated to play an anti-apoptotic role. Furthermore, survival rate of mice or cells was improved, MTD was prolonged, and HW/BW, viral titer and myocardial pathological score caused by viral infection were decreased. Studies (Huang et al., 2019; Zhang et al., 2019) also showed that H9c2 cells with myocardial ischemia (hypoxia-induced) after treatment with emodin (15 μM or 20 μM) had the effect of down-regulating caspase-3 and caspase-9. Regarding the role of emodin, Leung et al. (2020) also reported that emodin can regulate the kinase (ERK)-1/2 signaling pathway *in vivo* and *in vitro* by activating extracellular signaling, increasing the expression of Bcl-2 and glutamate transporter-1 (GLT-l), and inhibiting the activated level of caspase-3.

#### MiR-138

MicroRNA (miRNA) is essential in a variety of biological processes, including cell differentiation and proliferation, apoptosis, and metabolism (Brennecke et al., 2003; Chen et al., 2004). MiRNA deregulation often disrupts critical cellular processes, leading to the onset and progression of various human diseases. The identified miRNA can target various target genes, and the target miRNA can also contain multiple miRNA binding sequences. Currently, it have shown hope of miRNA-based therapies in treating human diseases (Zhang Y. et al., 2018; Cheng et al., 2019; Wang et al., 2019; Pottoo et al., 2020; Wu et al., 2020). Among them, miR-138 has been reported to protect cardiomyocytes from hypoxia-induced apoptosis (He et al., 2013; Xiong H. et al., 2016). Emodin (10 μM) has been demonstrated to inhibit hypoxia-induced injury. And it can significantly reduce the decline in cell viability and apoptosis caused by hypoxia in a dose-dependent manner (Lei et al., 2014; Fan et al., 2018). Subsequently, it was reported that emodin can protect cardiomyocytes by regulating miRNA expression (Hua et al., 2015; Xiang et al., 2017). Overexpression of miR-138 enhanced the protective effect of emodin, while miR-138 silencing weakened the protective effect of emodin on hypoxia-induced injury, suggesting that emodin played a protective effect on cardiomyocytes by upregulation of miR-138 (Rukov and Shomron, 2011; Zhang et al., 2019). In addition, in hypoxia-induced damaged cells, the overexpression of Mixed Lineage Kinase 3 (MLK3), which is synergistic with miR-138, inactivates the Sirt1/AKT and Wnt/wp-catenin pathways (He et al., 2013). The activation of Sirt1/AKT and Wnt/w1-catenin pathways plays an important role in anti-apoptosis (Lehwald et al., 2011). Zhang et al. (2019) showed that emodin (15 and 20 μM) inhibited the hypoxia-induced damage of H9c2 cells by up-regulated miR-138 and activating Sirt1/AKT and Wnt/wp-catenin pathways. It suggested that emodin may promote hypoxia-induced cell viability and inhibit cell apoptosis by de-regulating the expression of miR-138.

#### MiR-26a

MiR-26a plays a crucial role in the regulation of cardiomyocytes (Liu et al., 2016). Huang et al. found that the up-regulation of miR-26a promoted the apoptosis of hypoxia-treated H9c2 cells, while emodin (15 and 20 μM) could negatively regulate the expression of miR-26a and reduce the apoptosis by regulating miR-26a (Huang et al., 2019). Meanwhile, survivin is a member of the inhibitor of apoptosis protein (IAPs) family. It promotes cell survival by interfering with various cycle-related proteins, such as inner centromere protein and Aurora B kinase (Cheung et al., 2011). In addition, the relationship between hypoxic treatment and survivin expression has also been found in previous studies (Zhu et al., 2001; Zhang et al., 2007). Huang et al. (2019) revealed that hypoxia decreased the accumulated level of survivin while emodin enhanced the expression of survivin, which provided a reason for emodin to inhibit cell apoptosis due to the anti-apoptosis effects of survival. It is observed that MiR-26a achieves its functions *via* targeting survivin.

#### STAT3

It has been reported that signal transducer and activator of transcription (STAT)3 is an anti-apoptotic gene (Gu et al., 2020; Turovskaya et al., 2020). The activation of STAT3 may prevent cardiomyocyte apoptosis and enhance regulatory cardiovascular protective genes *in vivo* and *in vitro* during hypoxia-reoxygenation and I-R therapy (Mascareno et al., 2005; Ke et al., 2019). In a study (Huang et al., 2019), emodin can activate the inactivated JAK1/STAT3 pathway after hypoxia. Further results indicated that emodin (at the concentration of 15 and 20 μM) increased the phosphorylation of pathway-related proteins and that the JNK/STAT3 pathway inhibitor AG490 could weaken the protective effect of emodin on hypoxia-induced damage. This indicates that emodin alleviates hypoxia-induced cell damage through activation of the JAK1/STAT3 signaling pathway.

#### GSDMD

Myocardial I-R injury activates apoptosis and is accompanied by an inflammatory response (Toldo et al., 2018; Wu M. Y. et al., 2018). The TLR4/MyD88/NF-κB/NLRP3 inflammasome pathway is essential for activation of inflammation. Toll-like receptors recruit myeloiddifferentiationfactor88 (MyD88), leading to activation of NF-κB (Ve et al., 2017). NF-κB increases synthesis of NLRP3, pro-IL-1β, and IL-1β (Afonina et al., 2017). Activated NLRP3 inflammasome cleaves pro-caspase-1 to produce activated caspase-1 (Ives et al., 2015). GSDMD-FL could be cleaved by activated caspase-1 (canonical inflammasomes) or activated caspase-11 (non-canonical inflammasomes) (Jorgensen and Miao, 2015). Then, the GSDMD-N forms a large pore in the plasma membrane (Sborgi et al., 2016) and controls IL-1β release (Shi et al., 2015). Subsequently, water enters into the cell, causing cell swelling and eventual lysis (Shi et al., 2017). Therefore, GSDMD-N is the executioner of pyroptotic cell death. It has been reported that I-R injury could activate pyroptosis, which were at least partially mediated by the TLR4/MyD88/NF-κB/NLRP3 inflammasome pathway by Ye et al. (2019). Emodin (10 µM) inhibits the expression of TLR4 by decreasing ROS production and reduces the expression of GSDMD-N by decreasing the expression of TLR4, MyD88, NF-κB, and the NLRP3 inflammasome.

### Anti-Myocardial Fibrosis

#### MTA3

Cardiac fibrosis has been identified as the cause of heart failure (Jin et al., 2018). Emodin can improve multi-tissue fibrosis (Guan et al., 2016; Wang et al., 2016). Therefore, Xiao et al. (2019) evaluated the beneficial effects and potential mechanisms of emodin on cardiac fibrosis. Firstly, it was observed that emodin can reduce cardiac fibrosis by TAC-induced and cardiac fibroblast activation by AngII-induced. Then, it was found that the emodin/MTA3 axis play a crucial role in regulating cardiac fibrosis. This is consistent with the recognized role of MTA3 in regulating a variety of cellular events (Jiao et al., 2017). As a transcription factor, MTA3 becomes a cancer suppressor by inhibiting the proliferation, invasion, and migration of cancer cells (Ma et al., 2016). In addition, MTA3 also has anti-fibrosis effect (Qin et al., 2015). Xiao et al. (2019) further demonstrated that the upregulation of MTA3 may be molecular mechanisms of emodin inhibiting cardiac fibrosis.

### Anti-Cardiac Hypertrophy

#### HDAC

Lysine acetylation on histone tails is a reversible epigenetic modification that is regulated by two enzymes: histone deacetylases (HDACs) and histone acetyltransferases (HATs) (Yang and Seto, 2007). It has been reported that inhibition of class I and class II HDAC can effectively treat pathological myocardial hypertrophy in animal models (Antos et al., 2003). The pan-HDAC inhibitor TSA increases the acetylation of histones and attenuates cardiac hypertrophy (Yoshimura et al., 1993; Nakagawa et al., 1995). Recently, it has been reported that emodin can inhibit HDAC activity in a test tube (Godoy et al., 2017). *Levi W. Evans et al.* (Evans et al., 2020) has reported that emodin (10 μM) normalized cardiac gene expression with changes similar to the well-known HDAC inhibitor TSA. Emodin has been shown to block pathological cardiac hypertrophy *in vivo* and *in vitro*, which is consistent with its role as an HDAC inhibitor.

#### SIRT3

SIRT3 is a member of the Class III HDACs family (Zheng et al., 2019). It has been shown to play a key role in maintaining normal mitochondrial biological function through reversible deacetylation of protein lysine. Inhibition of SIRT3 can lead to defects in mitochondrial function, leading to the development of various cardiovascular diseases. The activation of SIRT3 may represent a promising therapeutic strategy to improve mitochondrial function and treat related cardiovascular diseases (Mihanfar et al., 2018; Wu J. et al., 2019). Gao et al. (2020) extracted rhubarb ingredients and cardiac hypertrophy targets based on machine learning and the network pharmacology method, and the optimal component-target combination (emodin-SIRT3) was obtained and further functional analysis was carried out on the combination. The results showed that emodin could treat or prevent agonist-induced and pressure-overload mediated cardiac hypertrophy. This suggested that activation of SIRT3 may be potential mechanisms for emodin to prevent mitochondrial dysfunction.

### Anti-Oxidative Damage

Emodin can act on the antioxidant system (Huang et al., 1991; Yi et al., 2004). Due to the ability of single electron transfer, emodin is considered as a generator of reactive oxygen species (Shi et al., 1994). Studies have showed that the formation of myocardial oxygen free radicals (oxygen free radicals) increased during I-R, and mitochondria were the main source of oxygen free radicals. Myocardial I-R injury was associated with decreased mitochondrial ATP production and antioxidant level/activity (Munro and Treberg, 2017; Ryoo and Kwak, 2018). The decrease of mitochondrial ATP production might be due to the oxidative damage of the protein complexes involved in the electron transfer process, while the consumption of non-enzymatic antioxidants, such as GSH and a-toc and the inhibition of antioxidant enzymes such as GRD and SOD will aggravate the oxidative damage of mitochondria (Yim et al., 1998). Du and Ko (2005) studied the sensitivity of emodin in a single dose treatment to mitochondrial ATP production and antioxidant composition in male and female rats and ischemia reperfusion (IR) injury. The results showed that a single dose of emodin can enhance the antioxidant components of mitochondria and prevent IR damage. Early experimental results have demonstrated the increased formation of oxygen-derived free radicals (oxy-radicals) in the myocardium during reperfusion after ischemia, wherein mitochondria are the major source of oxy-radical production. Pharmacological pretreatment of myocardium with low dose emodin chronic therapy can significantly enhance the antioxidant components of mitochondria. Du and Ko (2006) demonstrated that the combination of emodin in a low dose (25 μM/kg/day ×15) and IPC pretreatment produced a protective effect on the heart in a semi-additive manner. It protects the heart muscle from I-R damage.

### Anti-Proliferation

Atherosclerosis is the main cause of ischemic heart disease, and percutaneous coronary intervention is the main method to treat coronary artery occlusion (Fischman et al., 1994; Serruys et al., 1994). However, progressive arterial restenosis caused by abnormal proliferation and migration of vascular smooth muscle cells (VSMC) impedes effective treatment (Hao et al., 2003). Therefore, the concern is inhibition of migration and proliferation of vascular smooth muscle cells. It has been reported that emodin can inhibit the proliferation of VSMC, thereby inhibiting the hyperplasia of the intima (Xu et al., 2018a). It is reported by Wang X. et al. (2007) that emodin in a dose-dependent induced apoptosis of hVSMC and two additional forms of injury: autophagy and senescence. It may be regulated by ROS and p53 pathways. Subsequently, Wang S. J. et al. (2008) also proved that emodin directly inhibited VSMCs proliferation induced by Ang II in a dose-dependent manner. And inhibiting the expression of PCNA and c-myc may contribute to its anti-proliferative activity. Further evidence suggested that the proto-oncogene c-myc plays a key role in the cardiac protective properties of emodin.

### Other Functions

The cardiovascular protective effects of emodin are still under further exploration. Zhou et al. (2014) demonstrated that emodin (30 μM) increased ANP secretion via inhibition of L-type Ca(2+) channels through an activation of K(+)ATP channel in isolated beating rabbit atria. The results also provided role of emodin in the treatment of cardiovascular homeostasis. Wu et al. (2014) has reported that emodin may have great therapeutic potential for DCM in the treatment of diabetes-induced cardiomyopathy by Akt/gsk-3 signaling pathway. A study has demonstrated that emodin significantly prevented DEP induced cardiac inflammation and oxidative stress, and thrombotic complications *in vivo* and *in vitro* (Nemmar et al., 2015). This suggested that the use of functional food such as emodin can be considered a useful agent and may have the potential to protect people of all ages against the cardiovascular events in cities with high concentrations of particulate air pollution which was pending further studies.

## Potential Applications in Cardiovascular Diseases Therapy

Emodin targets multiple features of cardiovascular diseases through a series of pathways and has a variety of targets. Antioxidant, anti-viral, and anti-fibrotic effects are similar to conventional therapeutic methods or target protein inhibitors. In addition, it also showed good synergistic therapeutic effect. All these make emodin have broad application prospects in the treatment of cardiovascular diseases (**Table 2**). Among them, emodin has antioxidant properties. Pretreatment in Sprague-Dawley isolated mouse hearts (male and female) with I-R injury improved their antioxidant capacity, which was similar to the antioxidant a-tocopherol (a-TOC) and a-lipoic acid (a-LA) (Du and Ko, 2005). Emodin antiviral, reported by Liu et al. (2013) in BALB/c mice with viral myocarditis (CVB4), 15 mg/kg/d emodin treatment was equivalent to 10 mg/kg/d ribavirin can reduce viral cardiomyopathy. Antifibrosis role in hypertensive rats with LV fibrosis in Goldblatt (2K1C), Emodin, irbesartan or two drugs together can potentially inhibit the ventricular fibrosis in Goldblatt hypertensive rats. Furthermore, the combination of these two drugs may provide a better anti-fibrosis effect than the single application (Chen et al., 2014). In neonatal rat ventricular myocytes (NRVMs) with myocardial hypertrophy (phenylephrine induced), emodin blocks pathological cardiac hypertrophy and reverses stress-induced transcriptome changes in cardiomyocytes, consistent with HDAC inhibitor TSA (Evans et al., 2020). Emodin has a protective effect on myocardial I-R injury. In Sprague-Dawley isolated rat hearts with I-R injury, the protective effect of emodin pretreatment on myocardial I-R injury is consistent with that of ischemic preconditioning (IPC), and the protective effect of emodin and IPC combined pretreatment on myocardium is semi-additive (Du and Ko, 2006).

**Table 2:** Emodin and conventional therapeutic methods against cardiovascular disease.

Drug	Drug type	Disease model	Therapeutic effect comparison	References
A-tocopherol (a-TOC), a-lipoic acid (a-LA)	antioxidant	Sprague-Dawley isolated rat hearts (male and female) with I-R injury	Emodin pretreatment improved antioxidant capacity comparison to a-TOC or a-LA to a similar extent.	(Du and Ko, 2005)
Ischemic preconditioning (IPC)	Surgery	Sprague-Dawley isolated rat hearts with I-R injury	Emodin preconditioning protect against myocardial I-R injury consistent with IPC and combined emodin and IPC pretreatment produced cardioprotective action in a semi-additive manner.	(Du and Ko, 2006)
Ribavirin	Synthetic nucleoside	BALB/c mice with viral myocarditis (CVB4)	15 mg/kg/d emodin treatment was equivalent to 10 mg/kg/d ribavirin in alleviating the virus myocardial lesions.	(Liu et al., 2013)
Irbesartan	ATR1 blockers	hypertensive rats with LV fibrosis in Goldblatt (2K1C)	Emodin, irbesartan or two drugs together can potentially inhibit the ventricular fibrosis in Goldblatt hypertensive rats.	(Chen et al., 2014)
Trichostatin A (TSA)	HDAC inhibitor	Neonatal rat ventricular myocytes (NRVMs) with Myocardial hypertrophy (Phenylephrine induced)	Emodin blocked pathological cardiac hypertrophy and reversed stress-induced changes in the cardiomyocyte transcriptome consistent with TSA.	(Evans et al., 2020)

## Toxicity

Although emodin has beneficial pharmacology activities, it has been found that hepatotoxicity, nephrotoxicity, and reproductive toxicity limit its further study. First, it was found in studies that emodin has hepatotoxic effects (Zhang M. et al., 2018). Chen et al. (2020b) found that long-term or high-dose emodin showed a cytotoxic effect in hepatocytes and inhibited hepatocyte nuclear factor 4α expression, thereby reducing UDP-glucuronosyltransferase 2B7 (UGT2B7) expression and causing hepatotoxicity. Wu L. et al. (2018) also showed that there were gender differences in the hepatotoxicity and toxicology of emodin, which may be mediated by the coupling of UGT2B7 and multidrug-resistant-protein 2 *in vivo*. Second, with regard to the nephrotoxicity of emodin, the results of the United States National Toxicology Program showed that mice taking emodin at 22 mg·kg^−1^ were associated with tubular injury (increased incidence of tubular pigmentation) and increased incidence of kidney disease in female mice (National Toxicology Program, 2001). After 24 h of emodin was applied (40 and 80 µm), cell viability was significantly reduced by approximately 29% and 45%, respectively, which was mediated by the induction of apoptosis in the caspase 3‐dependent pathway and the mitochondrial pathway (Wang C. et al., 2007, Wang et al., 2015). Wang C. et al. (2008) have shown that emodin has cytotoxic effects on HK-2 cells, partially *via* induction of cathepsin B (CB) protein and activation of CB protease. And then, the CK2α’ knockout mice were given emodin orally for 5 days (1000 mg/kg/day), and it was found that emodin had testosterone toxicity due to disruption of testicular gene expression (Oshida et al., 2011). The different doses of emodin (25–400 μM) were applied to ejaculated human sperm, showed that sperm functions were inhibited by reducing sperm [Ca(2+)]i and prohibiting tyrosine phosphorylation *in vitro *(Luo et al., 2015). In addition, it was found that 24 h emodin (100 μM) interrupted several metabolites and biological processes including amino acid metabolism, purine metabolism, and Krebs cycle disruption (Chen et al., 2018). Since the ROS scavenging agent NAC can counteract the toxic effect of emodin, the increase in mitochondrial reactive oxygen production was also considered as an aspect of emodin toxicity (Kolitsida and Abeliovich, 2017).

## Pharmacokinetics

Emodin has a wide range of biological activities, but its bioavailability is low when taken orally (Zhang M. et al. (2018). Shia et al. (2010) reported that after intragastric administration at doses of 20 or 40 mg/kg, emodin rapidly underwent phase II metabolism to form its glucuronide, and the parent form of emodin was almost undetectable *in vivo*. Even at higher doses (82.4 mg/kg), the levels of emodin detected *in vivo* remained very low. The appearance of glucuronidation metabolism and oxidative conversion into rhein may be the main reasons for the extremely poor oral bioavailability of emodin (Liu et al., 2012; Li et al., 2017; Cheng et al., 2020). Li et al. (2019) revealed that the absorption of emodin was faster, and the effect was rapider when used in combination with aloe-emodin, rhein, chrysophanol, or physcion. Trans-2,3,5,4′-tetrahydroxystilbene-2-O-β-d-glucopyranoside (TSG) can promote the absorption of emodin and increase its plasma concentration to enhance the efficacy of emodin (Xing et al., 2019). A similar study revealed that piperine significantly increased the C_max_ and area under concentration‐time curve (AUC) of emodin and decreased the C_max_ and AUC of emodin glucuronide (Di et al., 2015). Therefore, the application of emodin is greatly limited, because of its poor intestinal absorption, rapid elimination and low bioavailability *in vivo* after oral administration. In future studies, it is important and urgent to improve oral bioavailability in clinical practice.

## Emodin Derivatives

In order to apply emodin safely and effectively, the research on emodin derivatives has been carried out continuously. Chen et al. (2016) reported that the emodin derivatives, madagascine, has been ameliorated vasospasm related cardiovascular diseases. It exerted vasodilatation effect through activating adenosine 5′-monophosphate (AMP)-activated protein kinase (AMPK), leading to the activation of eNOS in endothelium and inhibition of rho-associated protein kinase/myosine phosphatase target subunit 1(ROCK/MYPT1) in vascular smooth muscle (VSM). Furthermore, under the same experimental conditions, compared with emodin, it has higher biological activity and safety. Huang et al. (2014) provided evidence that PPemd26, an anthraquinone derivative, suppressed angiogenesis through inhibiting the vascular Endothelial Growth Factor Receptor 2 (VEGFR2) signaling pathway, suggesting that it is a potential drug candidate for the development of anti-angiogenic agents for angiogenesis-related diseases, including coronary heart disease. Ji et al. (2020) reported that haloemodin had strong antibacterial activity against Gram-positive bacterial strains. Compared with emodin, haloemodin more rapidly increases the bacterial plasma membrane permeability for potassium ions. More importantly, compared with emodin, halomodin binds to protein tighter and more stable. The results partially explain the stronger antibacterial activities of haloemodin over their parent nucleus, emodin. Chen et al. (2020a) disclosed that Aloe‐Emodin (AE) could alleviate high-fat diet/palmitic acid-induced cardiac inflammation *via* inhibition of the TLR4/NF-κB signaling pathway. Thus, AE may be a promising therapeutic strategy to prevent myocardial injury κB and p-P65l caused by hyperlipidemia. *Bai’s* findings suggested a novel pharmacological role of AE in high-fat diet-induced cardiac electrical remodelling (Bai et al., 2017). Yu et al. (2019) findings indicated that AE can protect against myocardial infarction *via* the upregulation of miR-133, inhibition of ROS production and suppression of caspase-3 apoptotic signaling pathway. Zhang et al. (2020) results revealed that inhibiting the activation of NLRP3 inflammasome and reducing the release of HMGB1 by promoting NLRP3 ubiquitination, thereby restoring the endothelial tight junction proteins and permeability, which indicated that AE exhibited immense potential therapeutic value in hypertension-related cardiovascular disease and the development of innovative drugs (**Table 3**).

**Table 3:** Anti-cardiovascular effects of emodin derivatives.

Derivatives	Chemical name	Application: Cell lines/model	Activity/mechanism(s) of action	References
PPemd26	1,8-dihydroxy-4,5-dinitroanthraquinone	In vitro and In vivo: HUVECs, MDA-MB-231 and HCT116 cells; Sprague-Dawley rats.	Suppresses angiogenesis *via* inhibiting VEGFR2 signaling.	(Huang et al., 2014)
Madagascine	3-isopentenyloxyemodin	In vitro and *in vivo*: cell HUVECs and HCASMCs; Male Wistar rats.	Vasodilatation through activating AMPK, leading to the activation of eNOS in endothelium and inhibition of ROCK/MYPT1 in vascular smooth muscle	(Chen et al., 2016)
Novel haloemodin (HEI2)	2,4-diiodoemodin (HEI2), 2-iodioemodin (HEI1), and 2,4,5-triiodoemodin (HEI3)	In vitro: bovine serum albumin; Staphylococcus aureus ATCC6538, Bacillus cereus ATCC10231, and Enterococcus faecalis ATCC 29212	Exhibits strong antibacterial activity against Gram-positive bacterial strains, including those generally resistant to drugs, such as MRSA and VRE.	(Ji et al., 2020)
Aloe‐Emodin	1.8-Dihydroxy-3-[hydroxymethyl]-anthraquinone	In vivo: male Wistar rats; cardiomyocytes were obtained from Sprague-Dawley neonatal rats	Against myocardial infarction *via* the upregulation of miR-133, inhibition of ROS production and suppression of caspase-3 apoptotic signaling pathway.	(Yu et al., 2019)
		In vivo and *in vitro*: male Wistar rats. H9C2 embryonic rat heart-derived cell line	Alleviate HFD/PA-induced cardiac inflammation *via* inhibition of the TLR4/NF-κB signaling pathway	(Chen et al., 2020a)
		In vivo and *in vitro*: male Wistar rats, rat ventricular myocytes	Prevents HFD-induced QT prolongation by repressing miR-1 and upregulating its target Kir2.1.	(Bai et al., 2017)
		In vivo and *in vitro*: C57BL/6J male mice; microvascular endothelial cells (MECs) line hemangioendothelioma (EOMA)	Inhibits NLRP3 inflammasome activation and decreases the release of HMGB1 by promoting NLRP3 ubiquitination, and thus restoring the endothelial tight junction proteins and permeability	(Zhang et al., 2020)

## Summary and Outlooks

In summary, it is reported that emodin has a wide range of anti-cardiovascular activities. It is involved in multiple molecular targets, such as inflammatory regulation, anti-apoptosis, anti-hypertrophy, anti-myocardial fibrosis, inhibition of oxidative damage, and abnormal and excessive proliferation of smooth muscle cells. It showed a good therapeutic prospect in the treatment of myocardial hypertrophy, coronary heart disease, and myocardial ischemia/reperfusion injury. Numerous studies have also made huge strides in revealing the mechanisms behind these effects. In addition, similar therapeutic effects and better synergistic effects were also shown in studies comparing emodin with conventional therapeutic methods or target protein inhibitors. However, despite the encouraging results in this research field, the current research only exists in the animal research stage, and a systematic and overall understanding of its mechanisms of action has not yet been formed. More importantly, attentions should be paid to the toxicity of emodin due to the limitation of its use caused by its increased content and low bioavailability. They are the most important factor limiting in the future research. With the deepening of research on emodin, its toxicity has been continuously discovered, and it is necessary to search for more bioavailability and toxicity neutralization solutions.

## Author Contributions

Authors’ contributions were as follows: conception and design of the study (QL, JG, YW); combing the literature (QL, XP); drafting the article (QL); revisions for content (QL, AC); and critical revisions for important intellectual content (QL, YW). All authors contributed to the article and approved the submitted version.

## Funding

We acknowledge the financial supports from the National Natural Science Foundation of China (81573832), National Key Research and Development Program of China [2017YFC1700106], and Double First-Class Personnel Office-High Level Scientific Research Team Research Fund (1000061020100, 1000061223245).

## Conflict of Interest

The authors declare that the research was conducted in the absence of any commercial or financial relationships that could be construed as a potential conflict of interest.

## Abbreviations

AMI, acute myocardial infarction; BMP, bone morphogenetic protein; AngII, angiotensin II; ANP, atrial natriuretic peptide; CVB3, coxsackievirus B3; CVB4, coxsackievirus B4; EAM, experimental autoimmune myocarditis; eNOS, endothelial nitric oxide synthase; I-R, ischemia/reperfusion; IL-1β, interleukin-1β; IL-6, interleukin-6; IL-23, interleukin-23; IL-17, interleukin-17; ISO, isoproterenol; GSDMD, gasdermin D; GLT-l, glutamate transporter-1; HATs, histone acetyltransferases; HAECs, human aortic endothelial cells; HDACs, histone deacetylases; hVICs, human aortic valve interstitial cells; HSP, heat shock protein; JNK, jun kinase enzyme; MTA3, metastasis-associated protein 3; miR-223, microRNA-223; miR-138, microRNA-138; miR-26a, microRNA-26a; NO, nitric oxide; NF-kB, nuclear factor kappa-B; PPAR-γ, peroxisome proliferator-activated receptor-γ; MyD88, myeloiddifferentiationfactor88; TNF-α, tumor necrosis factor-a; TAC, transaortic constriction; VMC, viral myocarditis; VSMC, vascular smooth muscle cells; α-SMA, alpha-smooth muscle actin; PPAR-γ, peroxisome proliferator-activated receptor γ; SOD, superoxide dismutase; STZ, streptozotocin; STAT, signal transducer and activator of transcription; HSP90, heat shock proteins90; MLK3, mixed lineage kinase 3; AMP, adenosine 5′-monophosphate; AMPK, activated protein kinase; VEGFR2, vascular endothelial growth factor receptor 2; AE, aloe–emodin.
